# *Trypanosoma cruzi*-Infected Pregnant Women without Vector Exposure Have Higher Parasitemia Levels: Implications for Congenital Transmission Risk

**DOI:** 10.1371/journal.pone.0119527

**Published:** 2015-03-25

**Authors:** Victoria R. Rendell, Robert H. Gilman, Edward Valencia, Gerson Galdos-Cardenas, Manuela Verastegui, Leny Sanchez, Janet Acosta, Gerardo Sanchez, Lisbeth Ferrufino, Carlos LaFuente, Maria del Carmen Abastoflor, Rony Colanzi, Caryn Bern

**Affiliations:** 1 Duke Global Health Institute, Duke University, Durham, NC, United States of America; 2 Department of International Health, Johns Hopkins Bloomberg School of Public Health, Baltimore, MD, United States of America; 3 Laboratorio de Investigación en Enfermedades Infecciosas, Universidad Peruana Cayetano Heredia, Lima, Peru; 4 Universidad Catolica Boliviana, Santa Cruz, Plurinational State of Bolivia; 5 Hospital Universitario Japones, Santa Cruz, Plurinational State of Bolivia; 6 Department of Epidemiology and Biostatistics, University of San Francisco School of Medicine, San Francisco, CA, United States of America; Federal University of São Paulo, BRAZIL

## Abstract

**Background:**

Congenital transmission is a major source of new Trypanosoma cruzi infections, and as vector and blood bank control continue to improve, the proportion due to congenital infection will grow. A major unanswered question is why reported transmission rates from *T*. *cruzi*-infected mothers vary so widely among study populations. Women with high parasite loads during pregnancy are more likely to transmit to their infants, but the factors that govern maternal parasite load are largely unknown. Better understanding of these factors could enable prioritization of screening programs to target women most at risk of transmission to their infants.

**Methodology/Principal Findings:**

We screened pregnant women presenting for delivery in a large urban hospital in Bolivia and followed infants of infected women for congenital Chagas disease. Of 596 women screened, 128 (21.5%) had confirmed T. cruzi infection; transmission occurred from 15 (11.7%) infected women to their infants. Parasite loads were significantly higher among women who transmitted compared to those who did not. Congenital transmission occurred from 31.3% (9/29), 15.4% (4/26) and 0% (0/62) of women with high, moderate and low parasite load, respectively (χx2 for trend 18.2; p<0.0001). Twin births were associated with higher transmission risk and higher maternal parasite loads. Infected women without reported vector exposure had significantly higher parasite loads than those who had lived in an infested house (median 26.4 vs 0 parasites/mL; p<0.001) with an inverse relationship between years of living in an infested house and parasite load.

**Conclusions/Significance:**

We hypothesize that sustained vector-borne parasite exposure and repeated superinfection by *T*. *cruzi* may act as an immune booster, allowing women to maintain effective control of the parasite despite the down-regulation of late pregnancy.

## Introduction

Regional initiatives to control Chagas disease by eliminating blood-borne transmission and domestic vector infestation have had a remarkable impact on the burden of this neglected disease [1]. Estimates for *Trypanosoma cruzi* infection prevalence have fallen from 16–18 million in 1990 to 8 million in 2005 [2,3]. Nevertheless, a third or more of those infected are reproductive-aged women and girls who can transmit the parasite to offspring during successive pregnancies. In 1990, the vast majority of new *T*. *cruzi* infections were vector-borne, but in 2005, 26% of new infections were attributed to mother-to-child transmission; as vector and blood bank control continue to improve, the proportion due to congenital infection is expected to grow [3,4].

A major unanswered question is why reported transmission rates from *T*. *cruzi*-infected mothers vary so widely among study populations, ranging from 1% to >15% in published studies [5–14]. Studies have consistently shown that women with high parasite loads during pregnancy are more likely to transmit to their infants, but the factors that govern maternal parasite load are largely unknown [15–17]. Better understanding of this phenomenon could enable prioritization of screening programs to target women most at risk of transmission to their infants.

Congenitally infected infants are usually asymptomatic or have mild symptoms, but they have a 20–30% risk of chronic cardiac and/or gastrointestinal disease decades later [4]. Treatment is well tolerated and highly effective early in life, but many congenital infections go undiagnosed [15,18]. We studied congenital *T*. *cruzi* transmission in a cohort of women giving birth in an urban hospital in Bolivia, the country with the highest Chagas disease prevalence [4]. We evaluated factors associated with maternal parasite load during pregnancy and implications for congenital transmission.

## Materials and Methods

### Ethics statement

The protocol was approved by ethics committees of HUJ (protocol #006), Universidad Catolica Boliviana, Universidad Peruana Cayetano Heredia (UPCH) (protocol #56907), A.B. PRISMA, Centers for Disease Control and Prevention (protocol #5829) and Johns Hopkins Bloomberg School of Public Health (IRB #2644). All women provided written informed consent for their own and their infants’ participation.

### Study population and procedures

The study was conducted in Hospital Universitario Japones (HUJ) in the city of Santa Cruz. Although vector-borne *T*. *cruzi* transmission is absent in the urban area, the city receives many migrants from rural areas with intense transmission. Study nurses enrolled women presenting for delivery, obtained informed consent and collected demographic and epidemiological data, including a record of all houses each woman lived in throughout her life, duration of residence, construction materials and house infestation with the triatomine vector.

Maternal infection status was determined using commercial IgG serological assays. Blood was collected, centrifuged and screened by two rapid tests, TrypanosomaDetect (InBios, Seattle, WA) and Polychaco indirect hemaglutination assay (IHA; Lemos Laboratories, Santiago del Estero, Argentina) at a single dilution of 1:16. Sera were subsequently tested by IHA with multiple dilutions and Chagatest lysate ELISA, with Recombinante 3.0 ELISA as tie-breaker (both ELISAs from Wiener Laboratories, Rosario, Argentina). Positive results by 2 or more conventional tests defined confirmed infection [19].

In both maternal and infant blood clot specimens, quantitative real time PCR (qPCR) was conducted following published methods [15,20]. Standard phenol-chloroform DNA extractions were performed. The primer set Cruzi 1 (5'–ASTCGGCTGATCGTTTTCGA–3') and Cruzi 2 (5'–AATTCCTCCAAGCAGCGGATA–3') was used to amplify a 166 base-pair DNA fragment. The probe Cruzi 3 (5'–CACACACTGGACACCAA–3') was labeled with 5′FAM (6—carboxyfluorescein) and 3′MGB (minor groove binder). TaqMan Human RNase-P detection reagent (Applied Biosystems) was included as an internal control; results were considered valid only if the internal control was efficiently amplified. A non-template negative control was included in each run. PCR standard curves were generated by inoculating a blood clot specimen with 1 x 10^6^ *T*. *cruzi* Y strain trypomastigotes, followed by extraction and serial dilutions. The detection limit was determined to be 1 parasite/ml. A positive result was defined by a Ct value below the Ct value of the detection limit standard, which fell consistently between 37 and 38 cycles. The individual specimen parasite loads were calculated based on the standard curve included in batch run.

A study nurse attended the delivery of each rapid test-positive woman to collect cord blood. Venous blood was collected from infants of infected women at 30, 90, 180 and 270 days after birth. Infant specimens were evaluated using three techniques, the microhematocrit method, IgM immunoblots and qPCR as described above. The microhematocrit method is the standard method to diagnose congenital T. cruzi infection in the first months of life in Latin America [21,22]. Blood was placed in 4–6 heparinized microhematocrit tubes, sealed, and processed within 24 hours by centrifugation (12,000 rpm for 7 minutes) followed by microscopic examination. IgM Western blots were performed using trypomastigote excreted-secreted antigens (IgM TESA-blot) [23,24]. Ladder-like bands at 130–200 kDa on IgM TESA-blot demonstrate antibodies to Shed Acute Phase Antigens (SAPA), indicating acute or congenital infection [23]. Bands below 95kDa are considered non-specific. Sera from the 180 and 270-day specimens were analyzed by IHA and ELISA, as described above for maternal specimen testing.

A neonatologist managed infected infants following Bolivian Control Program guidelines, which require positive results by microhematocrit or serology at 6 to 9 months; treatment decisions were based on these assays. For this analysis, we considered an infant to have congenital infection if he/she had positive results by microhematocrit, positive serology at 6 months with ELISA absorbance value > 0.7, any positive serology at 9 months, positive PCR in 2 specimens collected at different time points or positive PCR plus positive IgM TESA-blot. In our earlier study, we found that 78% of uninfected infants still had positive results by Chagatest ELISA at 6 months but all absorbance values were <0.7 [15].

The significance of differences was tested by Wilcoxon signed rank, Kruskal-Wallis, χ^2^ or Fisher Exact test as appropriate. Spearman's rank correlations were performed. Analyses were conducted in Stata SE 12.0 and SAS 9.0.

## Results

A total of 596 pregnant women were enrolled from June 1, 2010 to October 31, 2011. Of these, 128 (21.5%) had confirmed *T*. *cruzi* infection. Compared to uninfected women, *T*. *cruzi*-infected women were older (median (interquartile range [IQR]): 28 (22–34) vs 22 (19–29), p<0.001), had higher parity (median (IQR): 3 (2–5) vs 2 (1–4), p<0.001) and higher percentage of cesarean births (54.7% vs 47.7%; p = 0.16). Infected women were less likely to have any secondary education (58% vs 74%; p = 0.006), more likely to have lived in an infested house (56% vs 25%; p <0.0001) and slightly more likely to have lived in a rural area (64% vs 57%; p = 0.17) than uninfected women.

Congenital transmission occurred from 15 (11.7%) of 128 infected women, including 3 (42%) of 7 women with twin births (p = 0.03 for twin vs singleton births). Three of 3 bichorionic twin births were associated with transmission compared to 0 of 4 monochorionic twin births (p = 0.29). In each twin birth, both infants were infected, bringing the total number of infected babies to 18. Four infants had ambiguous infection status: 2 infants with a single positive result by PCR who were subsequently lost to follow-up and 2 infants treated by the control program at 6 months based on IHA at a titer of 1:128 with ELISA absorbance of 0.2 in one case and negative ELISA result in the other. These mother-baby pairs were excluded from analyses of transmission risk. Among the 18 confirmed infected infants, diagnosis was based on micromethod in 4 (all positive by qPCR and TESA-blot as well) and qPCR in 12 (10 of whom also had positive IgM TESA-blot); two infants had high titer positive serology by two different assays at 6 months of age.

The transmission rate was higher from infected women with no reported vector exposure compared to those who ever lived in an infested house, but this difference did not reach statistical significance (Table 1). There was no significant difference in age between women who transmitted and those who did not. qPCR data were available for 120 infected women, of whom 55 (45.8%) had positive results with median load of 36.5 parasites/mL (IQR 22.2–80.8). Parasite loads were significantly higher among women who transmitted compared to those who did not and showed a strong dose-response relationship. Congenital transmission occurred from 31.3% (9/29), 15.4% (4/26) and 0% (0/62) of women with high, moderate and low parasite load, respectively (χ^2^ for trend 18.2; p<0.0001).

**Table 1:** Maternal factors associated with congenital *Trypanosoma cruzi* transmission risk.

Maternal factor	Congenital *T*. *cruzi* transmission n/N (%)	Odds ratio	P value
Parasite load (parasites/mL)^a^			
Low (< 1)	0/50 (0)	0.00	
Moderate (1–34)	4/38 (10.5)	0.26	
High (>35)	9/29 (31.0)	1.0	<0.0001
Age (years)			
13–19	2/15 (13.3)	1.23	
20–29	7/55 (12.7)	1.17	
30–46	6/54 (11.1)	1.0	0.92
Type of birth			
Twin	3/7 (42.9)	7.3	
Singleton	12/117 (10.3)	1.0	0.03
Lived in infested house			
Never	9/56 (16.1)	1.95	
Ever	6/67 (9.0)	1.0	0.28
Lives in infested house now			
No	14/104 (13.5)	2.96	
Yes	1/20 (5.0)	1.0	0.46
Years of living in infested house			
20 or more	0/22 (0)	0.00	
1–19	6/45 (13.3)	0.80	
None	9/56 (16.1)	1.0	0.049

The limit of detection for the quantitative PCR assay was 1 parasite/mL. Parasite load values below this cutoff were assumed to be 0 for the purpose of analysis.

^a^qPCR data were missing for 2 women who transmitted and 6 women who did not transmit to their infants. Four mother-infant pairs with ambiguous transmission status are excluded (see text).

Infected women without reported vector exposure had significantly higher parasite loads than those who had lived in an infested house (median 26.4 vs 0 parasites/mL; p<0.001) with an inverse relationship between years of living in an infested house and parasite load (Table 2). Parasite loads were lower among women who had ever lived in a house with thatch roof, mud walls or earth floors, materials associated with triatomine infestation. Housing characteristics had high correlations with each other (coefficients 0.79 to 0.86; p values <0.0001) and moderate but significant correlations with history of having lived in an infested house (coefficients 0.31 to 0.37; p values <0.0001). Mothers of twins had higher parasite loads than those with singleton births with borderline statistical significance. There were no significant differences in parasite load by maternal age or rural residence history.

**Table 2:** Factors associated with estimated parasite load in blood of *T*. *cruzi*-infected mothers at the time of delivery.

	Parasite load (parasites/mL)^a^				
Factor	>35	1–34	< 1	Median (IQR)	P value
Twin birth					
Yes	4 (57.1%)	1 (14.3%)	2(28.6%)	47.7 (0–96.5)	0.07^b^
No	25 (22.1%)	25 (22.1%)	63 (55.8%)	0 (0–32.2)	
Current house is infested					
Yes	1 (5.6%)	4 (22.2%)	13 (72.2%)	0 (0–1.4)	0.05^b^
No	28 (27.5%)	22 (21.6%)	52 (51.0%)	0 (0–46.4)	
Age (years)					
13–19	4 (36.4%)	2 (18.2%)	5 (45.5%)	0.16 (0, 141)	0.74^c^
20–29	14 (25.0%)	20 (35.7%)	22 (39.3%)	1.1 (0, 35.6)	
30–46	11 (22.0%)	16 (32.0%)	23 (46.0%)	0.4 (0, 32.2)	
Median (IQR) age	25 (21, 34)	29 (24, 34)	29 (22, 34)		0.40^c^
Lived in infested house					
Ever	9 (13.9%)	11 (16.9%)	45 (69.1%)	0 (0–9.2)	0.0002^b^
Never	20 (37.0%)	15 (27.8%)	19 (35.2%)	26.4 (0–64.1)	
Median (IQR) years	0 (0–7.0)	0 (0–16.0)	12.5 (0–20.5)		0.0005^c^
Lived in house with thatch roof					
Ever	7 (14.0%)	9 (18.0%)	34 (68.0%)	0 (0–9.2)	0.007^b^
Never	22 (31.4%)	17 (24.3%)	31 (44.3%)	19.5 (0–62.0)	
Median (IQR) years	0 (0–0)	0 (0–13.0)	1.0 (0–13.0)		0.02^c^
Lived in house with mud walls					
Ever	7 (12.3%)	11 (19.3%)	39 (68.4%)	0 (0–9.2)	0.001^b^
Never	22 (34.9%)	15 (23.8%)	26 (41.3%)	19.9 (0–62.3)	
Median (IQR) years	0 (0–0)	0 (0–15.0)	5.0 (0–15.0)		0.004^c^
Lived in house with earth floor					
Ever	9 (15.0%)	10 (16.7%)	41 (68.3%)	0 (0–10.7)	0.002^b^
Never	20 (33.3%)	16 (26.7%)	24 (40.0%)	22.3 (0–62.1)	
Median (IQR) years	0 (0–7.0)	0 (0–15.0)	8 (0–16.0)		0.02^c^
Lived in rural area					
Ever	18 (26.9%)	24 (35.8%)	25 (37.3%)	4.2 (0, 51.7)	0.75^b^
Never	11 (28.2%)	10 (25.6%)	18 (46.2%)	0.2 (0, 46.4)	
Median (IQR) years	7 (0, 18)	11 (0, 16)	4 (0, 17)		0.80^c^

The limit of detection for the quantitative PCR assay was 1 parasite/mL. Parasite load values below this cutoff were assumed to be 0 for the purpose of analysis.

^a^qPCR data were missing for 2 women who transmitted and 6 women who did not transmit to their infants. Four mother-infant pairs with ambiguous transmission status are excluded (see text).

^b^Comparison of parasite load distributions by maternal characteristic by Wilcoxon signed rank test.

^c^Comparison of years of exposure by parasite load category by Kruskal-Wallis test.

## Discussion

In our data, as in previous studies [15–17], maternal *T*. *cruzi* load strongly predicted congenital transmission, with a remarkable dose-response relationship. However, ours is the first study to link absence of vector exposure to increased maternal parasite load, and by implication, congenital transmission risk. A study in Argentina demonstrated the highest transmission rate in women from never-infested areas, with a lower rate in those from previously infested areas and the lowest rate in those from areas with ongoing infestation, but parasite load was not measured [25]. The authors hypothesized that in areas without vector-borne transmission the circulating parasite population may be enriched with strains better adapted to transplacental transmission [25]. In contrast, a study in Bolivia reported more frequent positive hemocultures in women living in highly infested municipalities, suggesting a positive relationship between parasite loads and current vector exposure; however, the study did not quantify parasite load by qPCR, and vector data were reported at the municipality level only [26]. In our data, a woman’s cumulative years of living in an infested house was a stronger predictor of parasite load than current home infestation. We found a significant inverse dose-response relationship between years of residence in an infested house and parasite load.

We hypothesize that sustained vector-borne parasite exposure and repeated superinfection by *T*. *cruzi* may act as an immune booster, allowing women to maintain effective control of the parasite despite the down-regulation of late pregnancy [17,27]. Mothers of congenitally infected infants have been shown to have lower interferon-γ production and decreased T-cell and monocyte activation compared to infected women without transmission, both at the time of delivery and post-partum [17]. The association of twin births with higher parasite load and increased transmission risk may also be related to intensified down-regulation in mothers carrying twins [28].

Parasite genetic differences are hypothesized to contribute to the wide variation in reported congenital transmission rates [29,30], but no strain differences have been detected between women who transmitted to their infants and those that did not [16,31,32]. However, little variation was seen in the genotypes infecting the women in these studies, and nearly all available data come from the Southern Cone, where *T*. *cruzi* II, V and VI are predominant [33,34]. Data from areas where *T*. *cruzi* I is the predominant genotype are too sparse to enable analysis of risk based on parasite lineage [35,36]. Furthermore, molecular tools with higher discriminatory power may be needed to adequately address this issue.

The low sensitivity of standard early diagnostic methods for congenital Chagas disease is a major challenge to control programs and research in the field. Microhematocrit in a single neonatal specimen likely misses more than 50% of infected infants [37,38]. Serology in late infancy has high sensitivity, but follow-up rates are often below 20% [18,39]. While PCR has much higher sensitivity than micromethod for early diagnosis, the technique requires a well equipped laboratory and experienced personnel, and is beyond the resources of most control programs. In research studies, infants not diagnosed early in life may be misclassified as uninfected if specimens are not examined at 6–9 months by serology. Heterogeneity of follow-up may therefore contribute to differences in apparent transmission rates between study sites [37]. The relatively low number of congenital transmissions limited our statistical power to show associations. Future investigations would be helpful to track vector exposure more precisely, measure parasite load and immune responses at different times during pregnancy and augment congenital transmission data to provide more statistical power.

Our data suggest that cumulative vector exposure may be a determinant of *T*. *cruzi* blood levels in pregnant women, with an inverse relationship between years of exposure and parasite load. Although it is impossible to assess entomological parameters for exposures that occurred in the past, the prevalence of domestic infestation and *T*. *cruzi* infection in domestic vectors in southern Bolivia has been extremely high, even in recent years [40]. In our study, 44% of infected women and 60% of those who transmitted to their infants reported never having lived in an infested house. Although it cannot be proven, many of these women may have been themselves infected congenitally. Successful vector elimination will not eliminate risk for the remaining infected women and their infants [41]. Vigilance in screening for congenital transmission must be maintained in regions that have eliminated vector-borne *T*. *cruzi* transmission and in non-endemic countries, such as the United States and Spain, with infected immigrants.
